# Identification of key biomarkers and immune infiltration in the synovial tissue of osteoarthritis by bioinformatics analysis

**DOI:** 10.7717/peerj.8390

**Published:** 2020-01-17

**Authors:** Weisong Cai, Haohuan Li, Yubiao Zhang, Guangtao Han

**Affiliations:** Department of Orthopedics, Renmin Hospital of Wuhan University, Wuhan, China

**Keywords:** Osteoarthritis, Synovial, Immune infiltration, Bioinformatics analysis

## Abstract

**Background:**

Osteoarthritis (OA) is the most common chronic degenerative joint disease and is mainly characterized by cartilage degeneration, subcartilage bone hyperplasia, osteophyte formation and joint space stenosis. Recent studies showed that synovitis might also be an important pathological change of OA. However, the molecular mechanisms of synovitis in OA are still not well understood.

**Objective:**

This study was designed to identify key biomarkers and immune infiltration in the synovial tissue of osteoarthritis by bioinformatics analysis.

**Materials and Methods:**

The gene expression profiles of GSE12021, GSE55235 and GSE55457 were downloaded from the GEO database. The differentially expressed genes (DEGs) were identified by the LIMMA package in Bioconductor, and functional enrichment analyses were performed. A protein-protein interaction network (PPI) was constructed, and module analysis was performed using STRING and Cytoscape. The CIBERSORT algorithm was used to analyze the immune infiltration of synovial tissue between OA and normal controls.

**Results:**

A total of 106 differentially expressed genes, including 68 downregulated genes and 38 upregulated genes, were detected. The PPI network was assessed, and the most significant module containing 14 hub genes was identified. Gene Ontology analysis revealed that the hub genes were significantly enriched in immune cell chemotaxis and cytokine activity. KEGG pathway analysis showed that the hub genes were significantly enriched in the rheumatoid arthritis signaling pathway, IL-17 signaling pathway and cytokine-cytokine receptor interaction signaling pathway. The immune infiltration profiles varied significantly between osteoarthritis and normal controls. Compared with normal tissue, OA synovial tissue contained a higher proportion of memory B cells, naive CD4+ T cells, regulatory T cells, resting dendritic cells and resting mast cells, while naive CD4+ T cells, activated NK cells, activated mast cells and eosinophils contributed to a relatively lower portion (*P* > 0.05). Finally, the expression levels of 11 hub genes were confirmed by RT-PCR.

**Conclusion:**

The hub genes and the difference in immune infiltration in synovial tissue between osteoarthritis and normal controls might provide new insight for understanding OA development.

## Introduction

Osteoarthritis (OA) is the most common chronic degenerative joint disease and is mainly characterized by cartilage degeneration, subcartilage bone hyperplasia, osteophyte formation and joint space stenosis (Mathiessen & Conaghan, 2017). Its clinical manifestations are chronic pain, tenderness, swelling, malformation, and stiffness of the joint. It affects the lives of 240 million people around the world and seriously threatens human health. According to the World Health Organization, approximately 9.6% of men and 18% of women over 60 years old suffer from OA, and 25% of OA patients have disabilities (Li et al., 2017). Therefore, it is essential to identify the cause of OA and find an effective treatment method. However, the etiology and pathogenesis of the disease are not completely understood. Many scholars believe that OA is caused by an imbalance between the degradation and synthesis of articular cartilage, extracellular matrix and subcartilage bone (Keen et al., 2015). However, a recent study showed that synovitis is also characterized as an important pathological change of OA.

Although the sequence of joint tissue lesions in OA has not yet been determined, researchers have observed imaging, pathological and clinical evidence that pathological changes in the synovium occur earlier than those in the cartilage in OA. The findings from several arthroscopy, MRI and ultrasound techniques have confirmed the existence of synovial morphological changes (thickening, edema) in early OA (Guermazi et al., 2011; Roemer et al., 2011; Scanzello et al., 2014). Synovial vascularization and hyperplasia have been observed since the early stage of OA. Leukocyte infiltration and cellulose deposition (mainly in the stage of chronic inflammation) occur in the synovium of OA at different stages, suggesting the existence of synovitis since the initial stage of OA (Deligne et al., 2015; Young et al., 2001; Mapp & Walsh, 2012). Despite the controversy, arthroscopic debridement of the joint cavity and synovium can effectively alleviate the pain symptoms of some OA patients, suggesting that there may be some specific stimuli that trigger synovial lesions and eventually lead to the pathophysiological changes of the whole joint into a vicious circle (Bratus-neuenschwander et al., 2018; Sellam & Berenbaum, 2010). However, there is little research on the molecular mechanism of why synovitis is able to promote the development of OA.

Bioinformatics is a new interdisciplinary subject combining molecular biology and information technology. It is of great significance to reveal the molecular mechanism of diseases (Raut, Sathe & Raut, 2010). As an emerging technology, gene chips have been used for the high-efficiency and large-scale acquisition of biological information, and the expression profile data of diseases can be extensively collected. In this paper, the data of OA and normal patients in the common gene chip database (Gene Expression Omnibus, GEO) were analyzed by bioinformatics tools. The immune infiltration in OA synovium was analyzed by performing the CIBERSORT algorithm method, which is widely used to assess the relative content of 22 kinds of immune cells (Ali et al., 2016; Xiong et al., 2018). The purpose of this study was to identify the key biomarkers of the abnormally expressed genes and immune infiltration in the synovium of osteoarthritis and to provide diagnostic and therapeutic targets for OA.

## Materials & Methods

### Microarray data

Gene expression profiles of GSE12021, GSE55235 and GSE55457 were downloaded from the GEO database [GPL96 platform, Affymetrix Human Genome U133A Array]. GSE12021 contains 19 samples, including 9 synovial tissue samples from normal joints and 10 synovial tissue samples from OA joints. GSE55235 contains 20 samples, including 10 synovial tissue samples from normal joints and 10 synovial tissue samples from OA joints. GSE55457 contains 20 samples, including 10 synovial tissue samples from normal joints and 10 synovial tissue samples from OA joints. R software (version 3.4.0; https://www.r-project.org/) and Bioconductor packages (http://www.bioconductor.org/) were used in the data analyses. The computer codes used in this study can be found in the Supplemental File s. The sva package (Leek et al., 0000) that contains functions for removing batch effects and other unwanted variation in high-throughput experiments were used for the normalization of GSE12021, GSE55235 and GSE55457.

### Identification of DEGs

The linear models for microarray data (LIMMA; http://www.bioconductor.org/packages/release/bioc/html/limma.html) package in Bioconductor was used to identify DEGs by comparing the expression values between synovial tissues from normal joints and those from OA joints. The corresponding *P* value of the gene symbols after t test were used, and adjusted *P* < 0.05 and —logFC—>2 were used as the selection criteria. The pheatmap package (https://bioconductor.org/packages/release/bioc/html/heatmaps.html) was used to draw a heatmap of the DEGs in R software.

### Gene Ontology (GO) and KEGG pathway analysis of DEGs

GO is a major bioinformatics tool for annotating genes and analyzing their biological processes. KEGG is a database resource for understanding high-level functions and biological systems from large-scale molecular datasets generated by high-throughput experimental technologies. The clusterProfiler package (https://bioconductor.org/packages/release/bioc/html/clusterProfiler.html) in Bioconductor was used to perform the GO and KEGG pathway analysis of the DEGs. *P* < 0.05 was considered statistically significant.

### PPI network construction and module analysis

STRING (Search Tool for the Retrieval of Interacting Genes/Proteins) was applied for predicting the PPI network and detecting the possible relationships (confidence score 0.4, maximum number of interactors = 0). In addition, the Molecular Complex Detection (MCODE) plugin in Cytoscape version 3.6.1 was used to screen modules of the PPI network in Cytoscape (degree cutoff = 2, node score cutoff = 0.2, k-core = 2, and max. depth = 100).

### Immune infiltration by CIBERSORT analysis

The CIBERSORT algorithm was used to analyze the normalized gene expression data obtained before, and the proportions of 22 kinds of immune cells were obtained. These immune cells included naive B cells, memory B cells, plasma cells, CD8+ T cells, naive CD4+ T cells, resting memory CD4+ T cells, activated memory CD4+ T cells, follicular helper T cells, regulatory T cells (Tregs), gamma delta T cells, resting NK cells, activated NK cells, monocytes, M0 macrophages, M1 macrophages, M2 macrophages, resting dendritic cells, activated dendritic cells, resting mast cells, activated mast cells, eosinophils and neutrophils. The samples were screened according to *p* value <0.05, and the percentage of each kind of immune cell in the samples was calculated. Principal component analysis (PCA) was performed to determine whether there was a difference in immune cell infiltration between the synovial tissue of OA patients and that of normal controls. The different immune infiltration levels of each immune cell between the two groups was analyzed by the vioplot package in R version 3.6.0.

### RT-PCR validation of the hub genes

To confirm the findings from the bioinformatics analysis, synovial tissue from 6 patients without OA and 9 patients with OA were harvested for RT-PCR validation. The study protocol was approved by the Ethics Committees of Renmin Hospital of Wuhan University (approval number: 2019K-K011), and all patients signed the informed consent. Total RNA from synovial tissue was extracted with TRIzol reagent (Invitrogen, Thermo Fisher Scientific, Inc.). RNA samples from total RNA were reverse transcribed to cDNA, and RT-PCR was carried out using the Revert Aid First Strand cDNA Synthesis Kit (Fermentas, USA). GAPDH was used as an internal reference. Relative mRNA expression was calculated using the 2-ΔΔCt method. One-way analysis of variance was used for the statistical analysis, and *P* < 0.05 indicated a significant difference.

## Results

### Identification of DEGs

**Figure 1:**
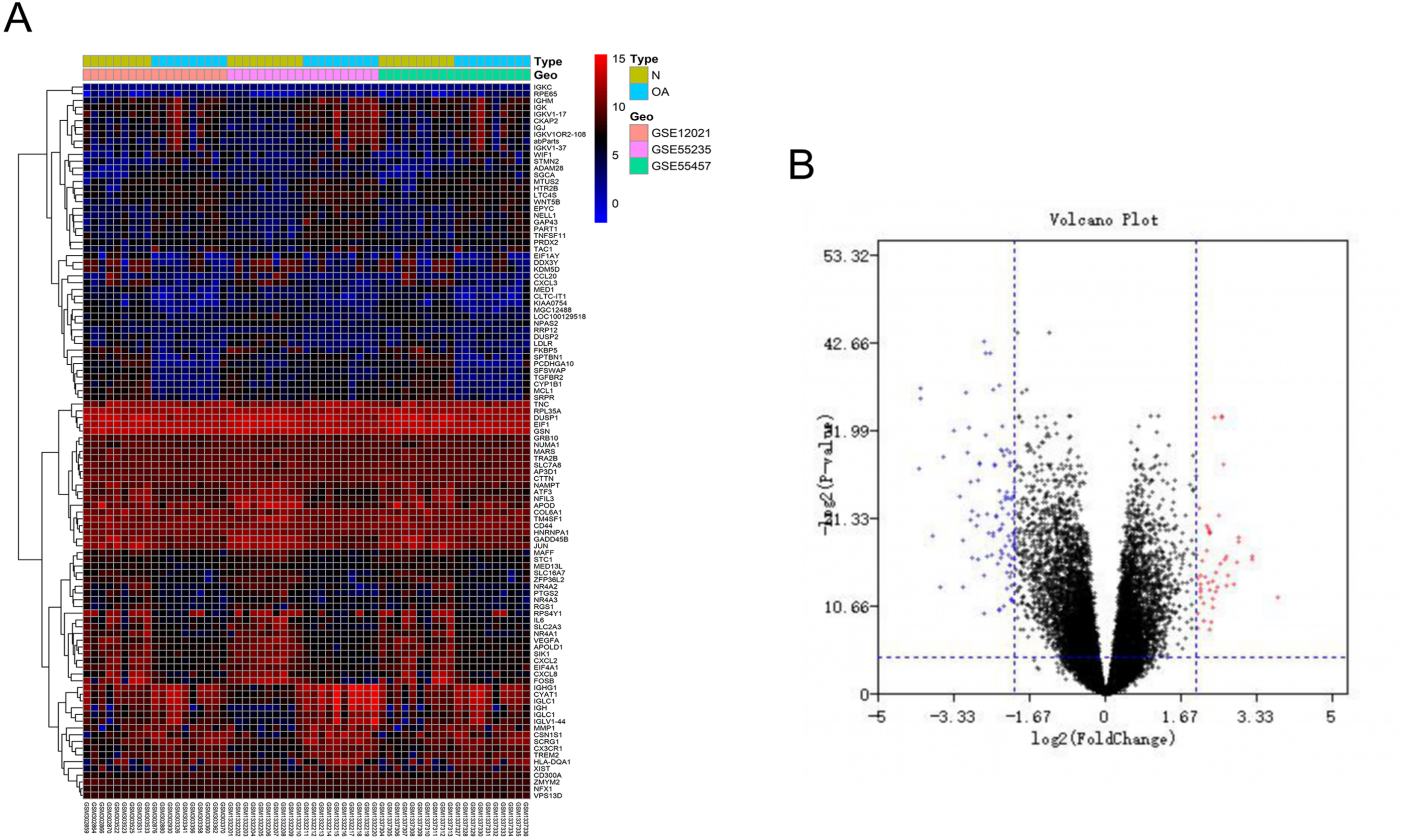
Differentially expressed gene expression heatmap and volcano plot between osteoarthritis and normal controls. (A) Differentially expressed gene expression heatmap of synovial tissue (all upregulated and downregulated genes). (B) Differentially expressed genes were selected by volcano plot filtering (adjusted *P* < 0.05 and | logFC| > 2).

After batch correction and standardization of the microarray results from GSE12021, GSE55235 and GSE55457, DEGs were identified. A total of 106 differentially expressed genes including 68 downregulated genes and 38 upregulated genes were detected. The results were validated with a volcano plot of all downregulated genes and upregulated genes (Fig. 1B). Figure 1A shows the DEG expression heatmap.

### GO function and KEGG pathway enrichment analysis of the DEGs

**Figure 2:**
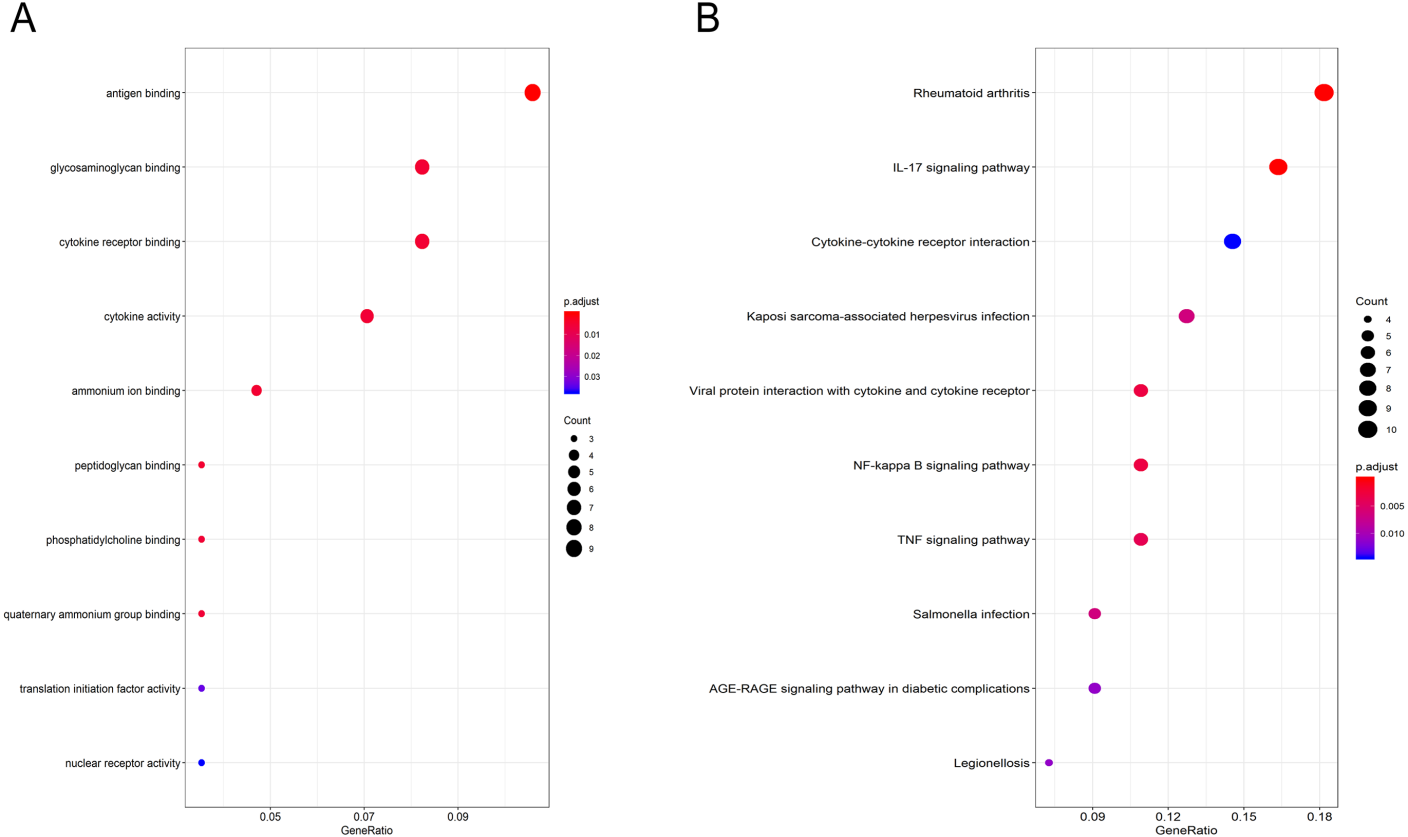
GO and pathway analyses results of DEGs between osteoarthritis and normal controls. (A) GO analyses results of DEGs. (B) Pathway analyses results of DEGs (top 10 according to adjusted *P* value).

Gene Ontology (GO) functional enrichment analysis showed that the DEGs were mainly involved in antigen binding, peptidoglycan binding, glycosaminoglycan binding, cytokine receptor binding, ammonium ion binding, cytokine activity, phosphatidylcholine binding, quaternary ammonium group binding, translation initiation factor activity and nuclear receptor activity (Fig. 2A, Table 1). KEGG pathway analysis revealed that the DEGs were mainly enriched in the IL-17 signaling pathway, rheumatoid arthritis, viral protein interaction with cytokines and cytokine receptors, the TNF signaling pathway, Salmonella infection, Kaposi sarcoma-associated herpesvirus infection, Legionellosis, the NF-kappa B signaling pathway, the AGE-RAGE signaling pathway in diabetic complications and cytokine-cytokine receptor interaction (Fig. 2B, Table 2).

**Table 1:** GO analyses results of DEGs (top 10 according to adjusted *P* value). “Count” means how many DEGs are involved.

ID	Description	P.adjust	Qvalue	Gene name	Count
GO:0003823	antigen binding	0.000	0.000	DQA1/IGHG1/IGHM/IGJ/IGKC/IGKV1-17/IGLC1/IGLV1-44/SLC7A8	9
GO:0005539	glycosaminoglycan binding	0.005	0.004	CD44/EPYC/IGHM/IGJ/TGFBR2/TREM2/VEGFA	7
GO:0005126	cytokine receptor binding	0.005	0.004	CCL20/CXCL2/CXCL8/IL6/TGFBR2/TNFSF11/VEGFA	7
GO:0005125	cytokine activity	0.005	0.005	CXCL2/CXCL8/IL6/NAMPT/TNFSF11/VEGFA	6
GO:0070405	ammonium ion binding	0.005	0.004	HTR2B/IGHM/IGJ/RPE65	4
GO:0042834	peptidoglycan binding	0.005	0.004	IGHM/IGJ/TREM2	3
GO:0031210	phosphatidylcholine binding	0.006	0.005	IGHM/IGJ/RPE65	3
GO:0050997	quaternary ammonium group binding	0.006	0.005	IGHM/IGJ/RPE65	3
GO:0003743	translation initiation factor activity	0.033	0.028	EIF1/EIF1AY/EIF4A1	3
GO:0004879	nuclear receptor activity	0.037	0.031	NR4A1/NR4A2/NR4A3	3

**Table 2:** Pathway analyses results of DEGs (top 10 according to adjusted *P* value). “Count” means how many DEGs are involved.

ID	Description	P.adjust	Qvalue	Gene name	Count
hsa05323	Rheumatoid arthritis	0.000	0.000	CCL20/CXCL2/CXCL3/CXCL8/HLA-DQA1/IL6/JUN/MMP1/TNFSF11/VEGFA	10
hsa04657	IL-17 signaling pathway	0.000	0.000	CCL20/CXCL2/CXCL3/CXCL8/FOSB/IL6/JUN/MMP1/PTGS2	9
hsa04060	Cytokine-cytokine receptor interaction	0.014	0.011	CCL20/CX3CR1/CXCL2/CXCL3/CXCL8/IL6/TGFBR2/TNFSF11	8
hsa05167	Kaposi sarcoma-associated herpesvirus infection	0.006	0.005	CXCL2/CXCL3/CXCL8/IL6/JUN/PTGS2/VEGFA	7
hsa04061	Viral protein interaction with cytokine and cytokine receptor	0.003	0.002	CCL20/CX3CR1/CXCL2/CXCL3/CXCL8/IL6	6
hsa04064	NF-kappa B signaling pathway	0.003	0.002	CXCL2/CXCL3/CXCL8/GADD45B/PTGS2/TNFSF11	6
hsa04668	TNF signaling pathway	0.004	0.003	CCL20/CXCL2/CXCL3/IL6/JUN/PTGS2	6
hsa05132	Salmonella infection	0.006	0.005	CXCL2/CXCL3/CXCL8/IL6/JUN	5
hsa04933	AGE-RAGE signaling pathway in diabetic complications	0.011	0.008	CXCL8/IL6/JUN/TGFBR2/VEGFA	5
hsa05134	Legionellosis	0.011	0.008	CXCL2/CXCL3/CXCL8/IL6	4

### PPI network construction and module analysis

**Figure 3:**
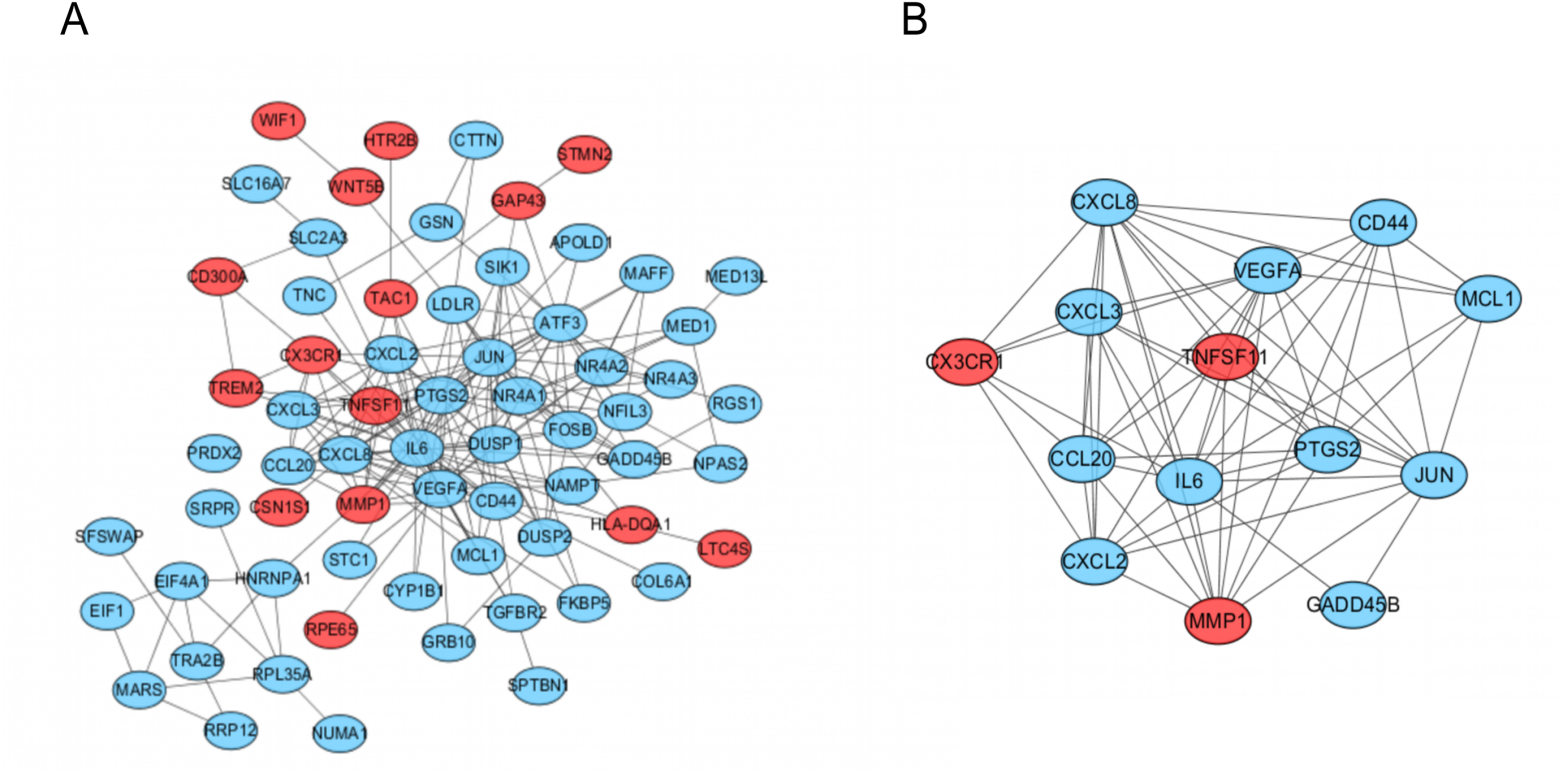
PPI network and the most signifcant module of DEGs between osteoarthritis and normal controls. (A) The PPI network of DEGs was constructed using Cytoscape. (B) The most signifcant module was obtained from PPI network with 14 hub genes. Upregulated genes are marked in red; downregulated genes are marked in blue.

The PPI network of the DEGs was analyzed by using STRING (Fig. 3A). The most significant module, including 14 hub genes, was obtained by using the MCODE plugin of Cytoscape (Fig. 3B).

### GO function and KEGG pathway enrichment analysis of the hub genes

**Figure 4:**
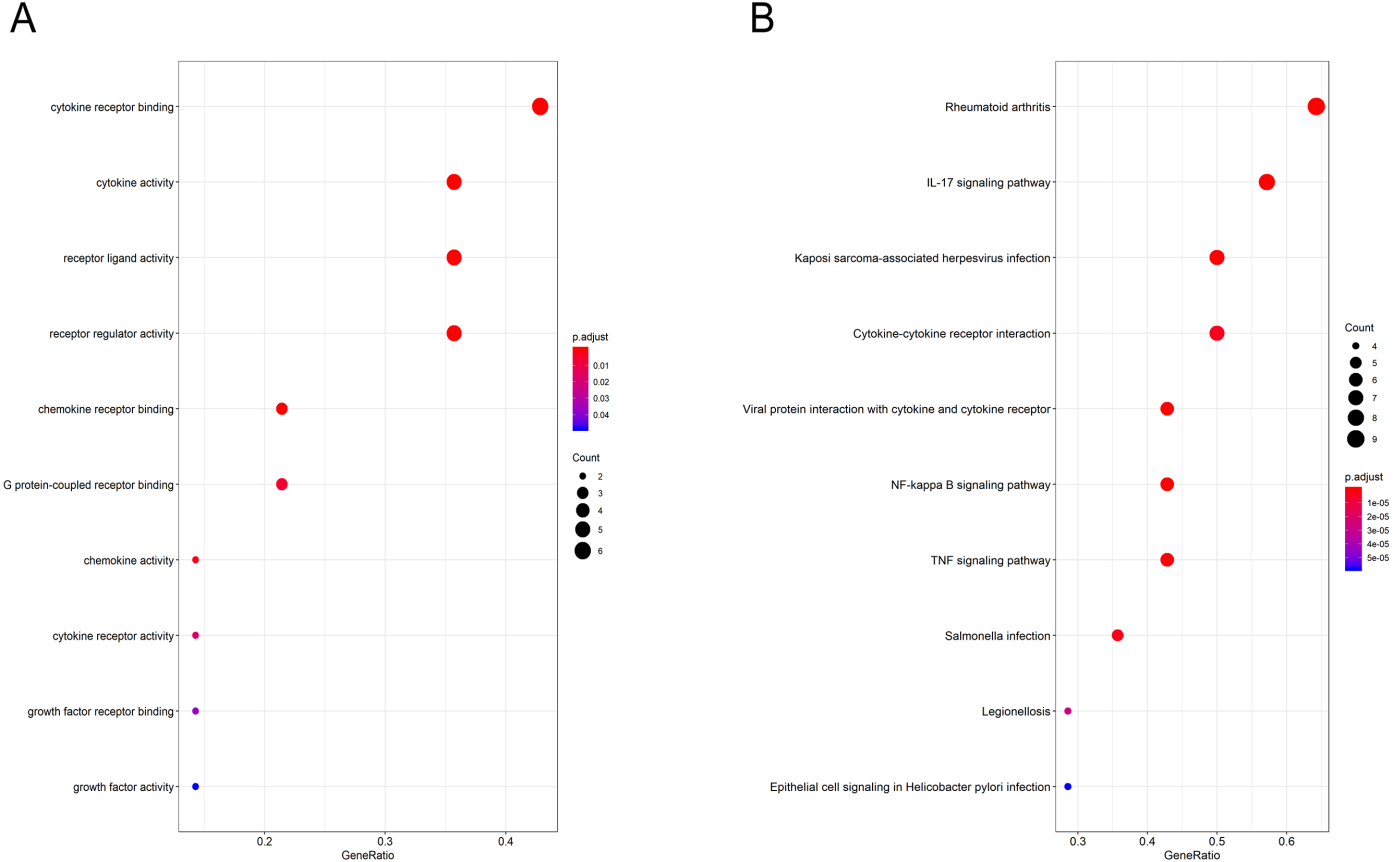
GO and pathway analysis results of hub genes between osteoarthritis and normal controls. (A) GO analyses results of hub genes. (B) Pathway analyses results of hub genes. (top 10 according to adjusted *P* value).

Gene Ontology (GO) functional enrichment analysis revealed that the hub genes were mainly involved in antigen binding, peptidoglycan binding, glycosaminoglycan binding, cytokine receptor binding, ammonium ion binding, cytokine activity, phosphatidylcholine binding, quaternary ammonium group binding, translation initiation factor activity and nuclear receptor activity (Fig. 4A, Table 3). KEGG pathway analysis showed that the hub genes were significantly enriched in rheumatoid arthritis, the IL-17 signaling pathway, Kaposi sarcoma-associated herpesvirus infection, cytokine-cytokine receptor interaction, viral protein interaction with cytokines and cytokine receptors, the NF-kappa B signaling pathway, the TNF signaling pathway, Salmonella infection, Legionellosis, and epithelial cell signaling in Helicobacter pylori infection (Fig. 4B, Table 4).

### Immune infiltration analyses

**Figure 5:**
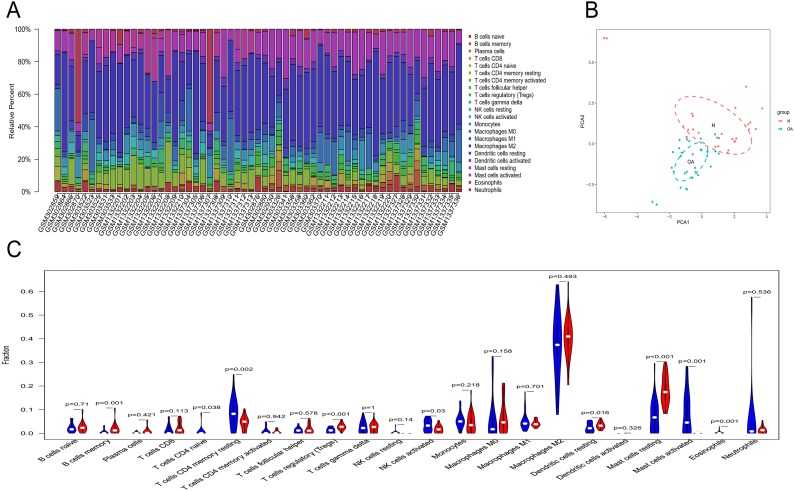
The landscape of immune infiltration between osteoarthritis and normal controls. (A) The relative percentage of 22 subpopulations of immune cells in 59 samples from GSE12021, GSE55235 and GSE55457 datasets.(B) Principal components analyses performed on all samples. The first two principal components which explain the most of the data variation are shown. (C) The difference of immune infiltration between osteoarthritis and normal controls.(The normal controls group was marked as blue color and osteoarthritis group was marked as red color. *P* values < 0.05 were considered as statistical significance.).

Owing to technical limitations, the landscape of immune infiltration in OA has not been entirely revealed, especially in subpopulations with a low abundance of cells. Using the CIBERSORT algorithm, we first investigated the difference in immune infiltration between OA and normal synovial tissues in 22 subpopulations of immune cells. Figure 5A summarizes the results obtained from 29 normal controls and 30 OA patients. By PCA, the proportions of immune cells from the tissues of OA patients and normal controls displayed distinct group-bias clustering and individual differences (Fig. 5B). Compared with normal tissue, OA tissue generally contained a higher proportion of memory B cells, naive CD4+ T cells, naive CD4+ T cells, regulatory T cells, resting dendritic cells and resting mast cells, whereas the proportions of naive CD4+ T cells, activated NK cells, activated mast cells and eosinophils were relatively lower (Fig. 5C, *P* < 0.05).

### RT-PCR validation of the hub genes

**Figure 6:**
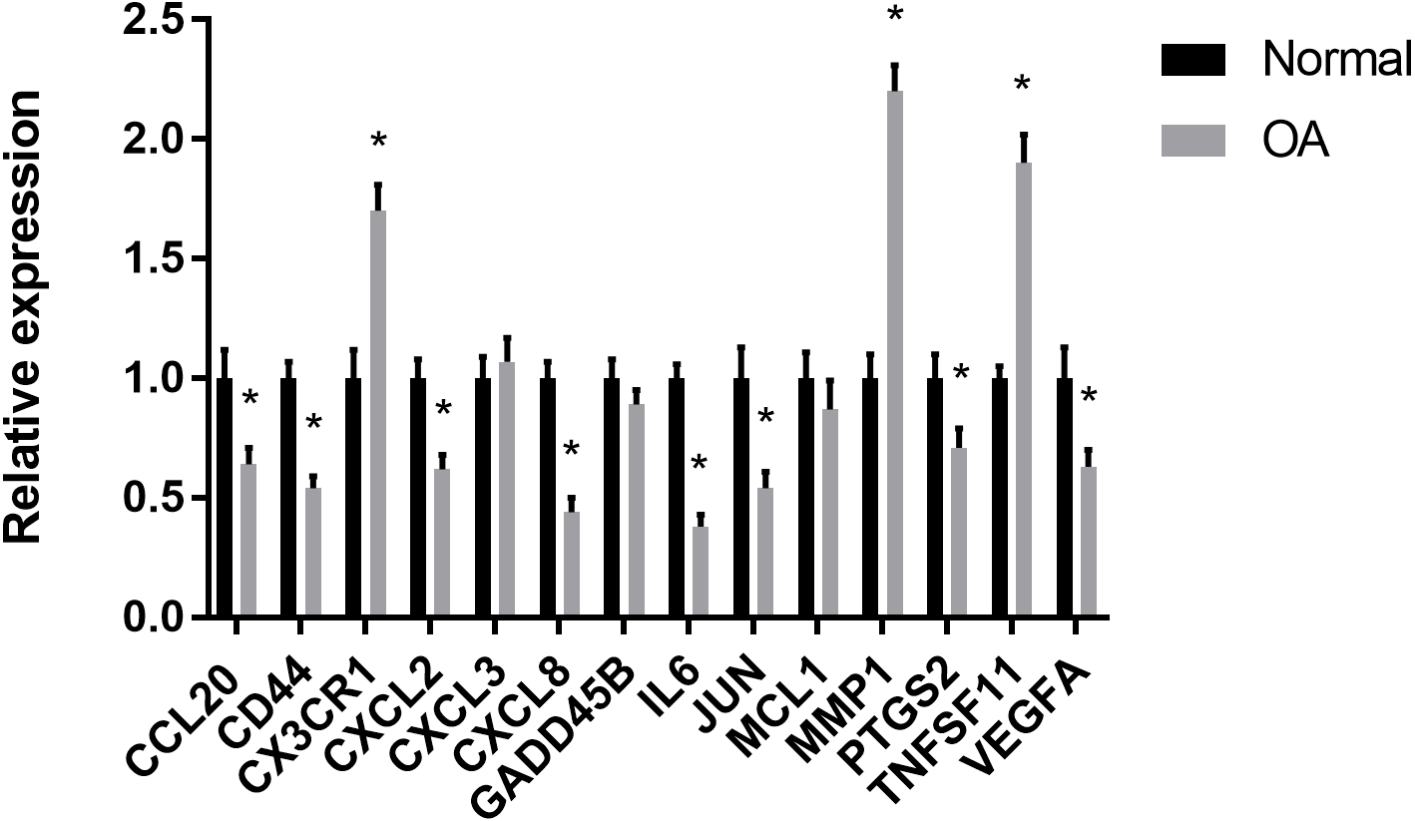
RT-PCR validation of the hub gene between OA and normal controls. All experiments were performed in triplicate and results were presented as M ± SD. (^∗^*p* < 0.05).

The results showed that the relative expression levels of 11 hub genes including CCL20, CD44, CX3CR1, CXCL2, CXCL8, IL6, JUN, MMP1, PTGS2, TNFSF11 and VEGFA were consistent with the microarray hybridization. CXCL3, GADD45B and MCL1 showed no statistically significant difference (Fig. 6).

## Discussion

Some of the previous OA studies emphasized only articular cartilage and ignored the role of soft tissue around the knee joint in the process of lesion development (Trachana et al., 2019). In recent years, increasing evidence has indicated that OA is accompanied by the occurrence and development of synovitis from the early stage to the late stage (Wang et al., 2018). Both acute and chronic synovitis can cause structural damage to bone and cartilage by forming inflammatory pannus, resulting in cartilage erosion and bone reconstruction, finally leading to secondary osteoarthritis (Ayral et al., 2005). Therefore, it is important to study the mechanism of synovitis in osteoarthritis. However, compared with rheumatoid arthritis, there are still few studies on the molecular pathological mechanism of synovitis in OA (Neumann et al., 2010). In this study, we tried to identify the important target genes related to synovitis in OA by comparing the differences in gene expression profiles between the synovium in osteoarthritis patients and that in normal controls. Fourteen key genes were screened out by bioinformatics analysis, and the biological functions and signaling pathways involved were analyzed in the present study. We found that these hub genes were involved in many immune responses and in immune cell chemotaxis, so we analyzed the immune cell infiltration of OA synovium by the CIBERSORT algorithm method, and the results showed that there was a significant difference in immune cell infiltration between OA synovium and normal controls.

**Table 3:** GO analyses results of hub genes (top 10 according to adjusted *P* value). “Count” means how many hub genes are involved,

ID	Description	P.adjust	Qvalue	Gene name	Count
GO:0005126	cytokine receptor binding	0.000	0.000	CCL20/CXCL3/CXCL8/IL6/TNFSF11/VEGFA	6
GO:0005125	cytokine activity	0.000	0.000	CXCL3/CXCL8/IL6/TNFSF11/VEGFA	5
GO:0048018	receptor ligand activity	0.000	0.000	CXCL3/CXCL8/IL6/TNFSF11/VEGFA	5
GO:0030545	receptor regulator activity	0.000	0.000	CXCL3/CXCL8/IL6/TNFSF11/VEGFA	5
GO:0042379	chemokine receptor binding	0.000	0.000	CCL20/CXCL3/CXCL8	3
GO:0001664	G protein-coupled receptor binding	0.006	0.003	CCL20/CXCL3/CXCL8	3
GO:0008009	chemokine activity	0.003	0.002	CXCL3/CXCL8	2
GO:0004896	cytokine receptor activity	0.018	0.009	CD44/CX3CR1	2
GO:0070851	growth factor receptor binding	0.037	0.019	IL6/VEGFA	2
GO:0008083	growth factor activity	0.049	0.025	IL6/VEGFA	2

**Table 4:** Pathway analyses results of hub genes (top 10 according to adjusted *P* value). “Count” means how many hub genes are involved.

ID	Description	P.adjust	Qvalue	Gene name	Count
hsa05323	Rheumatoid arthritis	0.000	0.000	CCL20/CXCL2/CXCL3/CXCL8/IL6/JUN/MMP1/TNFSF11/ VEGFA	9
hsa04657	IL-17 signaling pathway	0.000	0.000	CCL20/CXCL2/CXCL3/CXCL8/IL6/JUN/MMP1/PTGS2	8
hsa05167	Kaposi sarcoma-associated herpesvirus infection	0.000	0.000	CXCL2/CXCL3/CXCL8/IL6/JUN/PTGS2/VEGFA	7
hsa04060	Cytokine-cytokine receptor interaction	0.000	0.000	CCL20/CX3CR1/CXCL2/CXCL3/CXCL8/IL6/TNFSF11	7
hsa04061	Viral protein interaction with cytokine and cytokine receptor	0.000	0.000	CCL20/CX3CR1/CXCL2/CXCL3/CXCL8/IL6	6
hsa04064	NF-kappa B signaling pathway	0.000	0.000	CXCL2/CXCL3/CXCL8/GADD45B/PTGS2/TNFSF11	6
hsa04668	TNF signaling pathway	0.000	0.000	CCL20/CXCL2/CXCL3/IL6/JUN/PTGS2	6
hsa05132	Salmonella infection	0.000	0.000	CXCL2/CXCL3/CXCL8/IL6/JUN	5
hsa05134	Legionellosis	0.000	0.000	CXCL2/CXCL3/CXCL8/IL6	4
hsa05120	Epithelial cell signaling in Helicobacter pylori infection	0.000	0.000	CXCL2/CXCL3/CXCL8/JUN	4

The construction of protein–protein interaction networks, in which all protein-coding genes in a genome are grouped and organized, has been proven to be useful in the analysis of many kinds of diseases. By using MCODE in Cytoscape version 3.6.1, the PPI results were analyzed, and 14 hub genes, including CCL20, CD44, CX3CR1, CXCL2, CXCL3, CXCL8, GADD45B, IL6, JUN, MCL1, MMP1, PTGS2, TNFSF11 and VEGFA, were obtained in our study. And TNFSF11 was judged as the core gene according to the MCODE score. This gene encodes a member of the tumor necrosis factor (TNF) cytokine family which is a ligand for osteoprotegerin and functions as a key factor for osteoclast differentiation and activation (Mizoguchi, 2011; Ishii et al., 2011). Many studies have confirmed that osteoclasts, located at the pannus-bone junction and subcartilage bone, are important participants in the pathogenesis of bone destruction in patients with OA (Geurts et al., 2016). So TNFSF11 could be involved in OA progression by regulating the physiological function of osteoclasts. Furthermore, TNFSF11 can mediate the activation of the transcription factor NF-kB or MAPK, resulting in an inflammatory signal cascade waterfall response by binding to RANK (Walsh & Choi, 2014). In the animal model of arthritis induced by collagen or adjuvant, the level of serum TNFSF11 was significantly increased (Hairul-Islam et al., 2017). Similarly, some scholars have found that the level of TNFSF11 in synovial fluid increased in the serum and tissue of patients with RA (Hensvold et al., 2015). In our study, the expression of TNFSF11 also significantly increased in the synovium of OA patients. GO analysis also showed that this protein was involved in cytokine receptor binding, cytokine activity, receptor ligand activity and receptor regulator activity. It may also play an important role in the rheumatoid arthritis signaling pathway, cytokine-cytokine receptor interaction signaling pathway and NF-kappa B signaling pathway through KEGG pathway analysis. These data indicate that this gene plays a key role in OA progression and thus is useful as a therapeutic target.

Through GO analysis, we found that the hub genes were mainly involved in immune cell chemotaxis and cytokine activity. It is likely that leukocytic infiltration plays roles in the inflammatory reaction and catabolic enzyme production, leading to a disruption of joint tissue structure and function. However, many inflammatory molecules and immune cells also play a role in regulating the response to persistent tissue damage and can promote tissue repair attempts (Scanzello, 2016). In the present study, chemotactic factors and their related receptors, including CCL20, CD44, CX3CR1, CXCL2, CXCL3, CXCL8 and CCL20, were all differentially expressed in OA compared to normal controls by bioinformatics analysis. From the immune infiltration analysis, we also found that there was a significant difference in the relative cell content of memory B cells, naive CD4+ T cells, regulatory T cells, resting dendritic cells, resting mast cells, naive CD4+ T cells, activated NK cells, activated mast cells and eosinophils between the two groups. However, the concrete effects of these differentially expressed chemotactic factors on synovial immune infiltration need to be studied by further research. Apart from immune cell chemotaxis and cytokine activity, the hub genes including IL6 and VEGFA were also enriched in growth factor receptor binding and growth factor activity. A previous study showed that IL-6 is an important cytokine in the inflammatory reaction and is related to the degradation of cartilage matrix and the destruction of the synovium in OA (Pearson et al., 2017). VEGFA can promote angiogenesis by stimulating the mitosis of vascular endothelial cells and inducing the migration of endothelial cells. It can enhance and maintain the permeability of capillaries, which is not only beneficial to the formation of neovascularization in the form of budding but also conducive to the transport of nutrients and the diffusion of inflammation (Zupan et al., 2019). The proper level of VEGFA can improve the adaptation of synovial cells to cell injury caused by a hypoxic environment in the joint cavity. However, overexpression of VEGFA will lead to the abnormal proliferation of synovium and the aggravation of synovitis (Hamilton et al., 2016). Therefore, it is important to define the role of VEGFA in the synovium in the progression of OA.

Based on the KEGG database, 14 hub genes were mainly enriched in the rheumatoid arthritis pathway, IL-17 pathway and other inflammatory response pathways. The results indicated that OA was characterized by inflammatory processes in the process of disease. MMP1, a collagenase subfamily involved in the degradation of extracellular matrix, was upregulated in the rheumatoid arthritis pathway and IL-17 pathway in our study. As downstream genes of these two signaling pathways, overexpression of MMP1 can promote the degradation of cartilage proteoglycan and collagen and inhibit the formation of cartilage matrix (Wu, Du & Zheng, 2008). In a previous study, the content of MMP1 in the joint fluid of OA was significantly increased (Mahmoud et al., 2005). These results suggest that MMP1 may play an important role in the pathological process of OA formation. Our study also showed that this gene was upregulated in OA synovia based on the results of bioinformatics analysis and RT-PCR. However, another study showed that MMP-1 levels decreased with the progression of OA (Rübenhagen et al., 2012). Therefore, more studies are needed to define the function of MMP1 in the pathomechanism of OA. JUN is a transcription factor for activator protein 1 that mediates catabolic transcription and cell apoptosis/death and plays an important role in the IL-17 signaling pathway and TNF signaling pathway. A blockage of JUN could prevent chondrocyte degradation and apoptosis in OA (Chen et al., 2018; Ho et al., 2013; Chen et al., 2012). However, its downregulated expression could inhibit the apoptosis of synovial cells and promote synovial proliferation, resulting in synovitis (Lee et al., 2012). PTGS2, the key enzyme in prostaglandin biosynthesis, was enriched in the IL-17 signaling pathway, NF-kappa B signaling pathway and TNF signaling pathway through KEGG analysis. In a previous study, the expression of PTGS2 in joint fluid was related to the degree of pain in OA patients. A specific inhibitor of PTGS2 is widely used for the treatment of OA (Schneider et al., 2011; Scheiman, 2002). However, its gene expression in OA was downregulated compared with that in normal controls in our study. Many advanced OA patients experience chronic pain resistant to cyclooxygenase (COX) inhibitors (Sakurai et al., 2019). Another study also showed a lack of the chondroprotective effect of cyclooxygenase 2 inhibition in a surgically-induced model of osteoarthritis in mice. Its definitive role in the progression of OA still needs more research.

## Conclusions

In the present study, a total of 106 DEGs and 14 hub genes were identified. The expression levels of 11 hub genes were confirmed by RT-PCR. The biological functions and pathways of the identified genes provide a more detailed molecular mechanism for understanding OA development. By coupling reliable deconvolution algorithms with large-scale genomic data, we found a difference in immune infiltration between OA and normal controls. However, further studies are needed to confirm the relation between the key genes and immune infiltration and to define the functions of key genes and immune infiltration profiles in OA development.

##  Supplemental Information

10.7717/peerj.8390/supp-1 Supplemental Information 1 Code (batch correction and data normalization)

10.7717/peerj.8390/supp-2 Supplemental Information 2 Code (Identification of DEGs)

10.7717/peerj.8390/supp-3 Supplemental Information 3 Code (Cibersort analysis)

10.7717/peerj.8390/supp-4 Supplemental Information 4 Code (GEOimmune.CIBERSORT)

10.7717/peerj.8390/supp-5 Supplemental Information 5 GSE12021

10.7717/peerj.8390/supp-6 Supplemental Information 6 GSE55235

10.7717/peerj.8390/supp-7 Supplemental Information 7 GSE55457

## Additional Information and Declarations

### Data Availability

The following information was supplied regarding data availability: The data is available at NCBI GEO: GSE12021, GSE55235 and GSE55457. The code is available as Supplementary Files.
